# Investigating the tumor-immune microenvironment through extracellular vesicles from frozen patient biopsies and 3D cultures

**DOI:** 10.3389/fimmu.2023.1176175

**Published:** 2023-05-25

**Authors:** Ala’a Al Hrout, Mitchell P. Levesque, Richard Chahwan

**Affiliations:** ^1^ Institute of Experimental Immunology, University of Zurich, Zurich, Switzerland; ^2^ Department of Dermatology, University Hospital Zurich, University of Zurich, Zurich, Switzerland

**Keywords:** tumor microenvironment (TME), extracellular vesicle (EV), tumor immunity, immunotherapy, patient derived organoids

## Abstract

Melanomas are highly immunogenic tumors that have been shown to activate the immune response. Nonetheless, a significant portion of melanoma cases are either unresponsive to immunotherapy or relapsed due to acquired resistance. During melanomagenesis, melanoma and immune cells undergo immunomodulatory mechanisms that aid in immune resistance and evasion. The crosstalk within melanoma microenvironment is facilitated through the secretion of soluble factors, growth factors, cytokines, and chemokines. In addition, the release and uptake of secretory vesicles known as extracellular vesicles (EVs) play a key role in shaping the tumor microenvironment (TME). Melanoma-derived EVs have been implicated in immune suppression and escape, promoting tumor progression. In the context of cancer patients, EVs are usually isolated from biofluids such as serum, urine, and saliva. Nonetheless, this approach neglects the fact that biofluid-derived EVs reflect not only the tumor, but also include contributions from different organs and cell types. For that, isolating EVs from tissue samples allows for studying different cell populations resident at the tumor site, such as tumor-infiltrating lymphocytes and their secreted EVs, which play a central anti-tumor role. Herein, we outline the first instance of a method for EV isolation from frozen tissue samples at high purity and sensitivity that can be easily reproduced without the need for complicated isolation methods. Our method of processing the tissue not only circumvents the need for hard-to-acquire freshly isolated tissue samples, but also preserves EV surface proteins which allows for multiplex surface markers profiling. Tissue-derived EVs provide insight into the physiological role of EVs enrichment at tumor sites, which can be overlooked when studying circulating EVs coming from different sources. Tissue-derived EVs could be further characterized in terms of their genomics and proteomics to identify possible mechanisms for regulating the TME. Additionally, identified markers could be correlated to overall patient survival and disease progression for prognostic purposes.

## Graphical Abstract

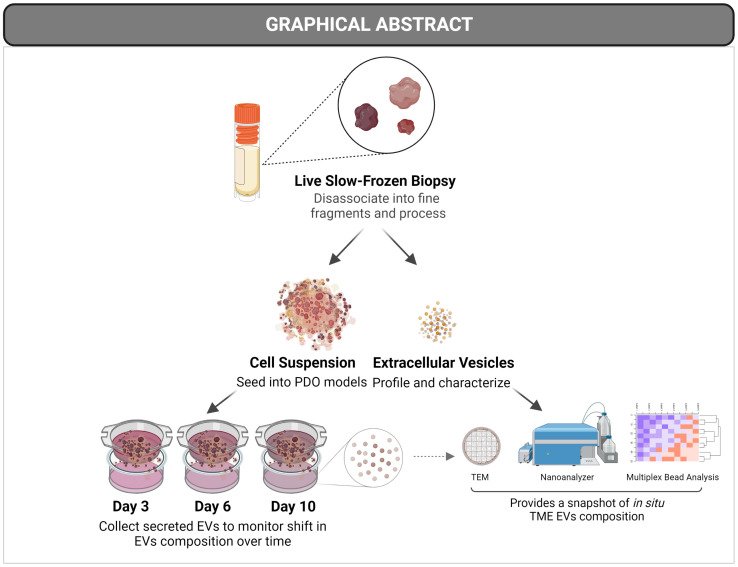

## 1 Introduction

### 1.1 Extracellular vesicles are powerful mediators of cell-cell communication

Cancer cells take part in a bidirectional interaction within their microenvironment. The tumor microenvironment (TME) is composed of a network of different types of cells, signaling molecules, and extracellular matrix. Non-malignant and immune cells can be recruited to promote a pro-tumor milieu and to sustain tumor growth and metastasis. The cross-talk within the TME is facilitated through the secretion of soluble factors, growth factors, cytokines, and chemokines. The release and uptake of secretory vesicles known as extracellular vesicles (EVs; also referred to as exosomes) has emerged as a powerful form of cell-cell communication. These vesicles range in size between 30-1000 nm and are secreted by different types of cells and have been studied intensively in the context of cancer where they’re implicated in promoting many cancer hallmarks (1). Through EV-based signaling, immune cells can be recruited to the tumor site to either promote an immune-activating or immune-suppressing outcome (2–5). EVs act as signaling cues by carrying internal and external biomolecules including RNA, proteins, and lipids (6, 7).

In the context of human patients, EVs are usually isolated from biofluids such as serum, urine, and saliva. Undoubtedly, such material provides valuable information about the patient and can be analyzed for diagnostic and/or prognostic purposes. Nonetheless, biofluid-derived EVs reflect not only the tumor, but also include contributions from different organs and cell types that secrete EVs that can reach the circulation. For that, tissue material represents a valuable resource to study EV-based signaling and TME tumor-immune interactions native to the tumor site. They can also be correlated to patient overall survival to identify prognostic markers.

### 1.2 Melanoma as a model for a better understanding of immune-tumor interactions and immunotherapy efficacy

Melanomas are highly immunogenic tumors as a result of their high genomic mutational load, where they have been shown to activate the immune response. Despite being the most promising cancer type for immunotherapy given the lack of effective alternative therapies, a significant portion of melanoma cases are either unresponsive or relapsed due to acquired immunotherapy resistance (8). During melanomagenesis, melanoma and immune cells undergo immunomodulatory mechanisms that aid in immune resistance and evasion. One mechanism by which melanoma cells escape destruction by immune surveillance is through presenting a less immunogenic phenotype, in addition to creating an immune-suppressive TME (9, 10). Melanoma-derived EVs have been implicated in immune suppression and escape, promoting tumor progression. Much like other tumor EVs, melanoma-derived exosomes package and shuttle protein and RNA to communicate with surrounding cells in their TME and modulate their function. Indeed, miRNAs are key players in melanomagenesis, where dysregulation in miRNA expression is linked to melanoma progression (11–14) and has been reported in different melanoma stages where they are utilized as biomarkers (15, 16). As such, miRNAs are essential contributors to the cross-talk within the TME and are thereby protected from degradation by shuttling them via different transporters, of which are exosomes (17). Melanoma exosomal miRNAs have been implicated in the metastasis of melanoma and the formation of a supportive tumor niche (18, 19). Melanoma also promotes immunotherapy resistance via overexpression of exosomal PDL-1, and not necessarily overexpressing cell-surface PDL-1 (20–22). Exosomal PDL-1 has been shown to elicit immunosuppressive effects, similar to tumors PDL-1, where blocking them induces systemic anti-tumor immunity (20, 21). Collectively, melanoma offers a promising platform to model and understand the role of immunity to cancer.

### 1.3 Three-dimensional organoid as an *ex vivo* model to study the tumor microenvironment

To reflect the complex nature of tumor biology, a physiologically relevant model that recapitulates not only the tumor but its TME is critical; especially in the context of drug discovery and identifying effective therapeutic targets. This is more pronounced in the field of immuno-oncology where promising therapies depend on the activation of cells in the TME, and equally, where the mechanism of therapy resistance is TME-driven. Available animal cancer models are unable to recapitulate the human TME faithfully, as most of these models lack immune cells, unless transplanted with a functional human immune system (23, 24), which proves to be problematic (Reviewed in (25). Nonetheless, the most widely used method to grow patient-derived cells is conventional two-dimensional (2D) cell culture, usually as an adherent monolayer on a plastic substrate. However, such an approach strips away many physiological parameters of the tumor. 2D cultures distort the physiological spatial arrangement of cells and hence, the cell-cell and cell-matrix interactions (26). It has also been shown that culturing cells as 2D cultures alter cellular response to therapy (27). Three-dimensional (3D) cell culture models provide physiologically relevant avenues for understanding *in vivo* context. Indeed, 3D cultured cells are shown to mimic *in vivo* architecture and gene expression profiles of tumors (26, 28). 3D cultures can be implemented in many different variations depending on the hypothesis to be tested. One of which is the spheroid/organoid model, which is a sphere formed from a microcellular cluster. Spheroids/organoids can form three distinct regions, a proliferative, a quiescent, and a hypoxic region creating a gradient of oxygen, nutrients, and exposure to drugs that mimic solid tumors *in vitro* (29–31). Patient-derived organoids (PDO) aid in modeling malignancy in a dish, under a controlled and modifiable environment, to answer a broad range of questions. PDO models based on co-culture or depletion of immune cells provide a platform to investigate the TME and help visualize the possible outcome of anticancer therapies. With the help of PDO models, we can also study the dynamic role EVs play in the TME in a more pointed direction and monitor the shift of TME signaling over time.

## 2 Materials and methods

Equipment list

ThermoMixer® C (eppendorf), or equivalent thermal mixer

Microcentrifuge

Ultracentrifuge

Reagents

-Tissue processing pipeline

Collagenase-IA (Sigma-Aldrich, USA, Cat# C2674-100MG)

Dispase-II (Sigma-Aldrich, USA, Cat# D4693-1G)

35 mm culture dish (Corning, USA, Cat# 430165)

Sterilized surgical scalpel #21 (Aesculap, Germany, Cat# BB521)

Cell strainer 70 um (Corning, USA, Cat# 431751)

10 mL syringe (B.Braun, Germany, Cat# 4606108v)

2 mL eppendorf tubes

Sterilized surgical forceps

-Patient-derived organoid media

GlutaMAX (Gibco, USA, Cat# 35050061)

B-27 Supplement without Vitamin A (Gibco, USA, Cat# 12587010)

N-Acetyl-L-cysteine (Sigma-Aldrich, USA, Cat# A9165-5G)

Nicotinamide (Sigma-Aldrich, USA, Cat# N0636-100G)

[Leu15]-Gastrin -I (Sigma-Aldrich, USA, Cat# G9145-.1MG)

Human EGF (PeproTech, USA, Cat# AF-100-15-100uG)

DMEM/Nutrient Mix. F-12 Ham (Sigma-Aldrich, USA, Cat# D8437-500ML)

50% L-WRN conditioned media (Prof. Martin Beaumont, GenPhySE France)

-Patient-derived organoid plates

10X HAM’S F10 (MP Biomedicals, USA, Cat# 91440049)

Cultrex collagen I (R&D Systems, USA, Cat#: 3440-100-01)

Reconstitution buffer (2.2 g NaHCO3 in 100 mL of 0.05 N NaOH and 200 mM HEPES) Neal et al., 2018

Millicell Culture Dish Insert (Fisher Scientific, USA, Cat#: PICM03050)

6-well plates

-Nano flowcytometry

PBS, pH 7.4 (Gibco, USA, Cat# 10010023)

FITC anti-human CD63 (Biolegend, USA, Cat# 353005)

Isotype control antibody (Biolegend, USA, Cat# 400201)

CellTrace™ Far Red (Invitrogen, USA, Cat# C34564)

Axygen® 0.6 mL MaxyClear Snaplock Microcentrifuge Tube (Axygen, USA, Cat# MCT-060-SP)

-Kits

EasySep™ Human CD19 Positive Selection Kit II (StemCell Technologies, Canada, Cat#: 17854)

MACSPlex Exosome Kit, human (Miltenyi Biotec, Germany, Cat#: 130-108-813)

### 2.1 Patient samples

Primary and metastatic melanoma flow-frozen human biopsies were obtained from the URPP biobank, Zurich. Collection, preparation, and freezing protocols of the fresh biopsies are outlined in (32). Informed consent had been obtained from all patients and all experiments were conducted according to the ethical rules of the Cantonal Ethic Committee of Zurich (Ethics form BASEC: 2014-0425).

### 2.2 Tissue sample processing

**Figure 1:**
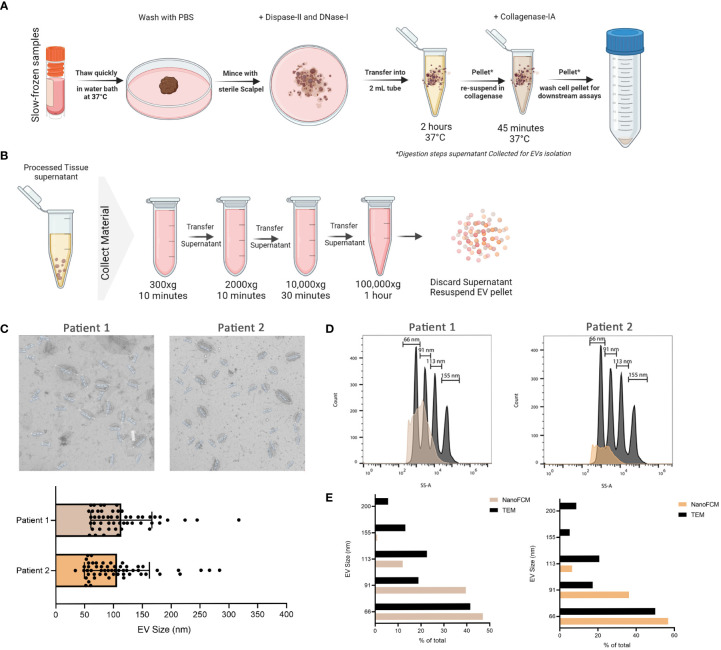
Isolation of EVs from frozen melanoma patient tissue. **(A)** Schematic representation of the tissue processing pipeline. Created with BioRender.com **(B)** Schematic representation of EVs collection pipeline. Created with BioRender.com **(C)** TEM representative images of 2 independent experiments and corresponding quantitative bar plot (mean ± SD). **(D)** Size estimation of tissue-derived EVs shown in C in comparison to silica sizing nanospheres (shown in dark grey) using nano-analyzer. **(E)** Comparison of EVs sizes obtained from TEM and nano-analyzer.

Cryovials containing slow-frozen biopsies were quickly thawed in a 37°C water bath and transferred into a BSL-2 hood onto ice. Tissue fragments are then collected with sterilized forceps and transferred into a pre-cooled 35mm dish. Tissue was then washed with chilled PBS and sliced with a sterile scalpel into smaller pieces in 1 mL of digestion enzyme mixture of Dispase-II (5mg/mL final concentration) and DNase-I (10ug/mL final concentration). Everything was collected into 2 mL eppendorf tube and the 35mm dish was washed with another 1 mL of the digestion enzyme mixture to collect any remaining sample and transferred to the 2 mL tube. Samples were incubated at 37°C with shaking in a heat-block for 2 hrs. After incubation, samples were centrifuged at 500xg for 5 mins to separate cells from EVs. The supernatant containing the EVs was collected into a fresh tube and kept at 4°C until analysis. The remaining cells were then incubated with 1 mL of Collagenase-IA (0.5 mg/mL final concentration) at 37°C with shaking in a heat-block for 45 mins. Samples were centrifuged at 500 x g for 5 mins to separate cells from EVs. The supernatant containing the EVs was collected into a fresh tube and kept at 4°C until analysis. The tissue processing pipeline is depicted in **Figure 1A**.

### 2.3 Patient-derived organoid models

After the two rounds of digestion, the cell pellet was washed with complete culture media and passed through a 70uM pre-wetted cell strainer. With a back of 5 mL syringe plunger, the remaining tissue was mildly dissociated, and an additional 5 ml of media was used to wash the cells through. Cells were centrifuged at 500 x g for 10 mins. PDO plates were prepared as described in (33). Briefly, the collagen gel matrix was prepared by diluting Cultrex Rat Collagen I (5mg/mL stock.) in 10X HAM and Sterile reconstitution buffer at a ratio of 8:1:1, on ice. Starting first with Cultrex Rat Collagen I and 10X HAM, after which reconstitution buffer was mixed in gently. Cell culture inserts were placed in a 6 well-plate and 1 mL of collagen gel matrix solution was added inside the insert, avoiding bubbles. The plate was incubated for 30 mins at 37°C to solidify. The single cell suspension is resuspended in 1 mL collagen gel matrix, carefully avoiding bubbles. Collagen-cell suspension is pipetted on top of the solidified 1 mL gel in the inserts. 2 mL of complete biopsy media is added to the outer well in the 6-well plate. The plate was placed in a 37°C incubator and kept for 10 days. Conditioned media was collected at days 3, 6, and 10 for EV isolation. For depletion experiments, Single cell suspension was divided into 2 tubes. One was depleted from CD19+ cells using StemCell EasySep™ Human CD19 Positive Selection Sample Kit II and seeded in the PDO system and referred to as the “depleted” condition. The other half was left without change and seeded as in the PDO system and referred to as the “complete” condition. Conditioned media was collected at days 6 and 10 for EV isolation (**Supplementary Figure 1A**).

### 2.4 EVs isolation and characterization

Digestion supernatant containing EVs was serially centrifuged to clear cells and debris. Briefly, the supernatant was centrifuged at 300 x g, followed by 2000 x g, for 10 mins each. The supernatant was then centrifuged at 10,000 x g for 30 mins before ultracentrifugation at 100,000 X g for 1 hr at 4°C. After ultracentrifugation, the supernatant was discarded and the EVs pellet was resuspended in PBS for downstream analysis. Pipeline is outlined in **Figure 1B**. For initial EV characterization, transmission electron microscopy (TEM) was used to visualize the morphology and size of EVs isolated from processed tissue samples. Briefly, samples were transferred onto pioloform-coated EM copper grids by floating the grids on a droplet containing freshly prepared exosome placed on parafilm and incubated for 5 mins. The grids were washed 3 times for 5 mins each before contrasting bound EVs with a mixture of 2% w/v methylcellulose and 2% w/v uranyl acetate (9:1 ratio) on ice for 10 mins. Grids were then allowed to air-dry before imaging. Sizes obtained from TEM were then compared to those obtained from nano-analyzer NanoFCM for the same samples. Using size reference beads on NanoFCM provided an overall size distribution in EVs samples.

### 2.5 EVs surface marker analysis

**Figure 2:**
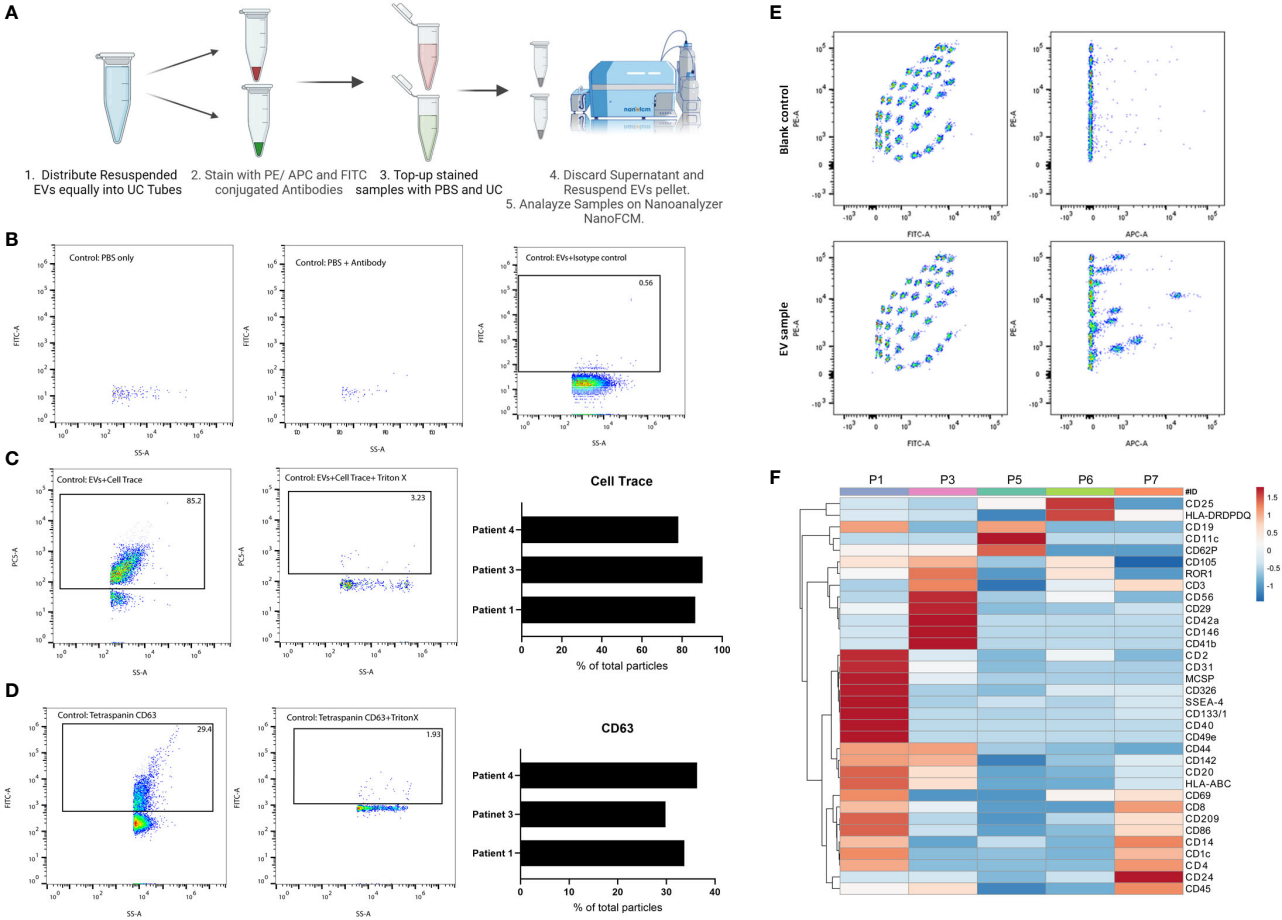
Characterization of tissue-derived EVs using single-particle and multiplex analysis. **(A)** Schematic representation of immunolabeling pipeline for nano flow-cytometry. Created with BioRender.com **(B)** FACS plot representatives of experimental controls (PBS, PBS-antibody, isotype control) on nano flow-cytometry. **(C)** FACS plot representatives of Cell Trace Far-Red positive particles and their corresponding control on nano flow-cytometry. 3 independent experiments are shown as a bar plot. **(D)** FACS plot representatives of CD63 positive particles and its corresponding control on nano flow-cytometry. 3 independent experiments are shown as a bar plot. **(E)** FACS plot representatives of multiplex analysis of tissue-derived EVs and corresponding blank control using Macsplex exosome kit. **(F)** Heatmap of expression of denoted surface markers of tissue-derived EVs from 5 independent experiments. Created with ClustVis.

To explore the EVs landscape in melanoma tissues, we looked into markers present on the surface of tissue-derived EVs using two approaches. The first approach uses a multiplex beads platform that provides an overview of the surface markers present through the detection of 37 broad markers simultaneously. Briefly, isolated EVs samples were incubated with EVs capture beads overnight and then washed. Bound EVs to beads were then incubated with EVs detection reagent for 1 hr at room temperature. Samples were washed and transferred to FACS tubes for downstream analysis. Blank control (beads and Macsplex buffer) was used to deduct the background signal (**Figure 2**).

The second approach to explore tissue-derived EVs surface markers provides a more targeted view through Immunostaining for specific markers and analysis on a nano flow-cytometer. Briefly, in 100 uL PBS, isolated EVs samples were incubated with antibodies against specific markers of interest for 1 hr on ice. After incubation, samples were topped with 1 mL PBS and then ultracentrifuged. The supernatant was discarded, and the EV pellets were resuspended in 50 uL PBS for downstream analysis. Experimental controls to exclude background noise and non-specific binding include PBS, Antibody solution in PBS, and isotype control stained EVs.

## 3 Results and discussion

### 3.1 EV isolation and characterization

To validate the successful isolation of EVs from frozen tissues, we used TEM as the gold standard of EV visualization and characterization. Based on our TEM analysis, the particles are round concaved bi-layer structures, and their sizes range between 50-180 nm, representing characteristics of EVs (**Figures 1A–C**). Size estimation was simultaneously carried out using the nano-analyzer NanoFCM by comparing detected particles to silica nanospheres cocktail of 4 different sizes (**Figure 1D**) as elaborated in our preprint (7). The size distribution of the particles is within the size range for small EVs. In addition, NanoFCM provided concentration estimation of tissue-derived EVs from patient 1 (8.47X10^9^ particles/mL) and patient 2 (3.77X10^9^ particles/mL). This variation is likely attributed to different starting materials analyzed since the volume of patient 1 biopsy was roughly half that of patient 2. NanoFCM-obtained sizes were compared to TEM-obtained sizes as TEM is considered the standard to characterize EVs (**Figure 1E**). NanoFCM was able to size the EV mixtures accurately in line with previous work (5, 34, 35), showcasing the different EVs subpopulation sizes. With that validation, we moved towards characterizing EVs through surface marker profiling.

It is worth noting that, to our knowledge, this is the first evidence showing the successful isolation of EVs from small frozen tissue biopsy samples. Recent work has demonstrated the possibility to isolate EVs from strictly fresh tissue collected within 1 hour of EVs isolation (36). However, this might prove to be challenging to implement as most clinical samples are obtained frozen from biobanks. Another advantage to analyzing frozen biopsies is that samples can be i) processed simultaneously thereby decreasing batch effects; ii) stored in multiple pieces allowing for technical repeats of experiments; and iii) can be batched together better after other classical experiments are carried out on other parts of the same biopsy.

### 3.2 EVs surface protein analysis from frozen tissue samples

In addition to isolating intact EVs, our method of processing the tissue preserved the surface proteins of EVs which allowed for surface markers profiling. We started with general quality controls to exclude background noise and non-specific binding (**Figures 2A, B**). As shown, a low number of particles were detected in PBS or antibody-PBS solution showcasing insignificant background noise. In addition, non-specific binding was also excluded through staining EVs with isotype control. Additional quality control was conducted using CellTrace Far Red (7), which will stain membranes and exclude non-biological particles and debris (**Figure 2C**). We observed an average of 85% of positive EVs for Cell Trace, denoting the high purity of our EV prep from frozen tissue samples. We were also able to detect CD63, an established marker for EVs, on the surface of EVs isolated from patients’ tissues (**Figure 2D**). With this approach, specific markers can be identified and quantified, which would not be possible with conventional flow cytometry or TEM approaches due to the limited size detection threshold or difficulty of co-staining, respectively. It also allows for analyzing EVs at the single particle level with respect to size, property, and marker distribution. The multiplex bead assay allowed for the detection of a range of markers from different patient’s tissue material, highlighting the enrichment of certain markers in one patient over another (**Figures 2E, F**). The multiplex bead assay demonstrated the enrichment of many different subtypes of EVs, including immune cell-derived EVs, reflecting the heterogeneous nature of the TME. Collectively, our data confirm the purity of our EVs prep derived from frozen tissue samples, and the possibility to characterize the different EVs present at the tumor site which provides a physiologically relevant snapshot of the TME.

### 3.3 Monitoring of EVs landscape over time

**Figure 3:**
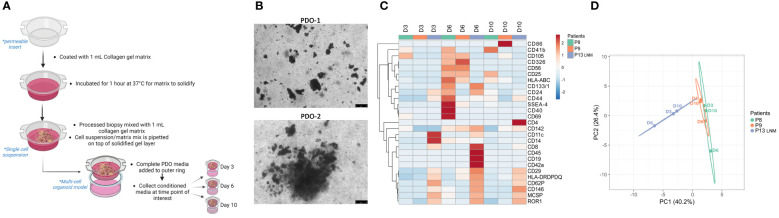
Characterization of PDOs-derived EVs using multiplex analysis. **(A)** Schematic representation of the PDO culture pipeline. Created with BioRender.com **(B)** Representative images of PDOs of 2 independent experiments at day 6 (scale bar= 75 µm). **(C)** Heatmap of expression of denoted surface markers of tissue-derived EVs from 3 independent experiments over 3,6, and 10 days. Created with ClustVis. **(D)** PCA plot of data shown in **(C)**. Unit variance scaling is applied to rows; SVD with imputation is used to calculate principal components. X and Y axis show principal component 1 and principal component 2 which explains 40.2% and 26.4% of the total variance, respectively. Created with ClustVis.

As a form of cell-cell communication, EVs provide a platform to monitor the changes in TME signaling over time. Another advantage of using PDO models to study EV-based communication lies in the flexibility of these models which allows for studying the specific contribution of a cell type. Single-cell suspension obtained from processed tissue samples were seeded in a PDO model for 3,6, and 10 days (**Figures 3A, B**). Secreted EVs were collected from 3 independent PDOs conditioned media and subjected to multiplex profiling. Based on the heatmap expression data, EVs surface proteins vary between different patients but also within the same patient when sampled from different days of PDOs culture (**Figure 3C**). Interestingly, day 6 PDOs-derived EVs seem to have a higher expression of markers amongst all the groups, which was also reflected in their clear separation on the PCA plot from other days of the same patient (**Figure 3D**). The PCA also demonstrated the closeness of patients 8 and 9 to each other in comparison to patient 13 (lymph node metastasis), reiterating the preservation of surface markers of tissue-derived EVs to the point of reflecting the TME at the time of sampling.

In addition, PDO models can also be utilized in exploring the contribution of a specific cell type to the EVs landscape in the TME. By depleting the cell population of interest prior to seeding the single-cell suspension as PDO models, one can monitor the shift in total EVs surface proteins over time (**Supplementary Figure 1A**). Single-cell suspension from one patient sample was divided into “complete” and “depleted” groups and seeded in a PDO model for 6 and 10 days. Secreted EVs were collected from PDO-conditioned media and subjected to multiplex profiling (**Supplementary Figure 1B**). Heatmap expression data highlighted the shift in EVs surface proteins expression between “complete” and “depleted” PDOs (**Supplementary Figure 1B**). Interestingly, groups seem to cluster based on the complete/depleted status rather than the day of culture. This was also reflected in the PCA plot where EVs derived from “complete” PDO cultures cluster on one side of the plot while EVs derived from “depleted” PDO cultures cluster on the other (**Supplementary Figure 1C**). With this approach, the depletion of a cell population of interest can be correlated to the over-/under- expression of certain EVs surface proteins, which could suggest a mode of action to this cell’s EV-based signaling.

Herein, we outline the first instance of a method for EV isolation from frozen tissue samples at high purity and sensitivity that can be easily reproduced without the need for complicated isolation methods. Our pipeline allows for the efficient recovery of tissue-derived EVs through a combination of enzyme cocktails, mechanical shaking, and ultracentrifugation. TEM imaging confirmed the isolation of intact EVs that were further characterized using nano flow-cytometry. The advantage of isolating EVs from tissue samples is that they provide a snapshot of the tumor and the interplay of the TME, which can’t be reproduced in cancer-derived cell lines that represent one population of the TME. Tissue-derived EVs also provide an insight into the physiologically relevant EVs enriched at the tumor site that can be overlooked when studying circulating EVs coming from different sources which can further dilute tumor-derived EVs. Isolating EVs from tissue samples allows for studying of different cell populations resident at the tumor site, i.e. TILs and their derived EVs, which play a central anti-tumor role. With our protocol, we demonstrated the successful isolation of EVs from frozen tissue which overcomes the need for freshly isolated tissue. Tissue-derived EVs could be further characterized in terms of their genomics and proteomics cargo to identify a possible mechanism for regulating the TME. Future applications could analyze adjacent-healthy tissue-derived EVs in comparison to their paired malignant tissue-derived EVs which can identify markers correlating to overall survival and disease progression for prognostic purposes.

## 4 Troubleshooting

• Mitigation 1: If the tissue sample didn’t disassociate properly after 2 rounds of digestion, then that might suggest that the tissue sample was sliced too coarsely.
– Solution: Slice each piece of tissue into finer fragments in subsequent experiments.
• Mitigation 2: If the cell yield is low after digestion, then it’s possible the cells adhered to the cell strainer.
– Solution: Pre-wet the strainer with cell culture media prior to passing cell suspension through, and wash with more media.
• Mitigation 3: If the number of viable cells is low, then this might suggest that the isolation was too slow.
– Solution: Work faster on ice while slicing the tissue sample.
• Mitigation 4: If PDO gel matrix is too viscous to pipette, then the gel matrix is starting to solidify.
– Solution: Keep all reagents chilled on ice, prepare gel matrix on ice, use pre-chilled pipette tips.
• Mitigation 5: If you observe spillovers when detecting EV markers on the nanoflow analyzer, then co-staining was done with overlapping fluorescently conjugated antibodies such as FITC and PE.
– Solution: co-stain with APC and FITC conjugated antibodies instead. Also prepare single stains to determine the percentage of spillover.
• Mitigation 6: If the background signal is too high on the nanoflow analyzer, that likely due to excess unbound antibodies.
– Solution: wash with PBS after staining and ultracentrifuge again, discard supernatant and resuspend in fresh PBS.

## Data availability statement

The original contributions presented in the study are included in the article/**Supplementary Material**. Further inquiries can be directed to the corresponding author.

## Ethics statement

The studies involving human participants were reviewed and approved by BASEC: 2014-0425. The patients/participants provided their written informed consent to participate in this study.

## Author contributions

AA and RC conceived the study. AA conducted the study and wrote the manuscript. RC provided funding, conceptual insights, and discussions. ML provided patient samples and insights. All authors contributed to the article and approved the submitted version.
