# Supplementary material for: Investigating the tumor-immune microenvironment through extracellular vesicles from frozen patient biopsies and 3D cultures
Source: Front Immunol. 2023 May 25;14:1176175. doi: 10.3389/fimmu.2023.1176175 (PMC10248017; doi:10.3389/fimmu.2023.1176175)
Supplement: Supplementary file 1 [file DataSheet_1.docx]

Supplementary Material

Investigating The Tumor-Immune Microenvironment Through Extracellular Vesicles From Frozen Patient Biopsies and 3D Cultures

Ala’a Al Hrout^1^, Mitchell P. Levesque ^2^, Richard Chahwan^1*^

^1^ Institute of Experimental Immunology, University of Zurich, Winterthurerstrasse 190, 8057, Zurich, Switzerland

^2^Department of Dermatology, University Hospital Zurich, University of Zurich, Zurich, Switzerland

*** Correspondence:** Richard Chahwan (chahwan@immunology.uzh.ch)

# Supplementary Data

Supplementary Material should be uploaded separately on submission. Please include any supplementary data, figures and/or tables.

Supplementary material is not typeset so please ensure that all information is clearly presented, the appropriate caption is included in the file and not in the manuscript, and that the style conforms to the rest of the article.

# Supplementary Figures and Tables

For more information on Supplementary Material and for details on the different file types accepted, please see [here](https://www.frontiersin.org/guidelines/author-guidelines#supplementary-material).

## Supplementary Figures


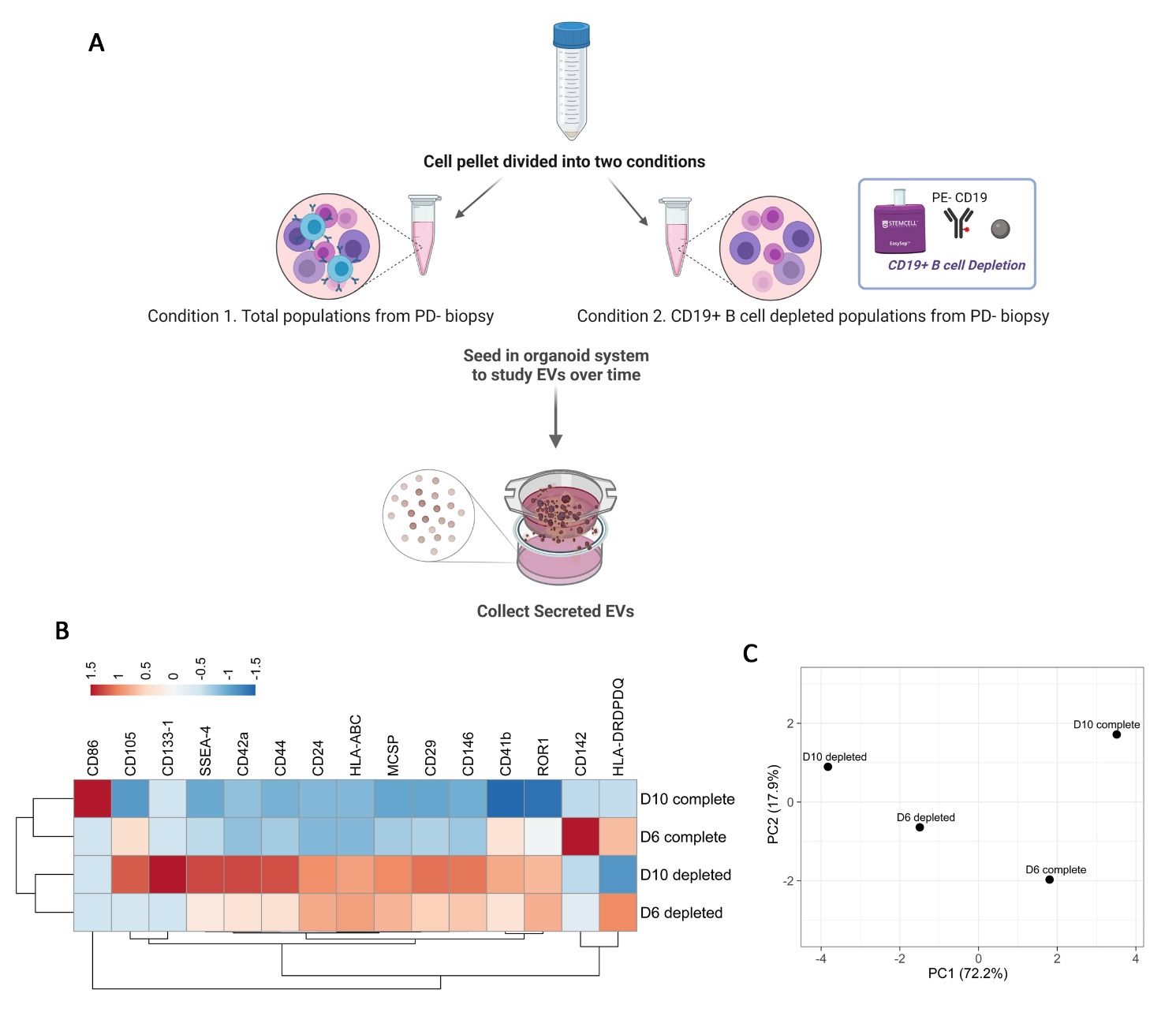


**Supplementary Figure 1.** (A) schematic representation of PDO depletion experiment pipeline. Created with BioRender.com (B) heatmap of expression of denoted surface markers of tissue-derived EVs from 1 experiment over 6 and 10 days. (C) PCA plot of data shown in B.
